# Prevalence and molecular subtyping of *Blastocystis* in patients with *Clostridium difficile* infection, Singapore

**DOI:** 10.1186/s13071-021-04749-8

**Published:** 2021-05-24

**Authors:** Lei Deng, Huiyi Tay, Guangneng Peng, Jonathan W. J. Lee, Kevin S. W. Tan

**Affiliations:** 1 Laboratory of Molecular and Cellular Parasitology, Healthy Longevity Translational Research Programme and Department of Microbiology and Immunology, Yong Loo Lin School of Medicine, National University of Singapore, Singapore, 117545 Singapore; 2 The Key Laboratory of Animal Disease and Human Health of Sichuan Province, College of Veterinary Medicine, Sichuan Agricultural University, 611130, Chengdu, Sichuan People’s Republic of China; 3 Department of Medicine, Yong Loo Lin School of Medicine, National University of Singapore, Singapore, 119228 Singapore; 4 Department of Gastroenterology and Hepatology, National University Health System, Singapore, 119074 Singapore

**Keywords:** *Blastocystis*, *Clostridium difficile*, Pathogenicity, Diarrhea, ST7

## Abstract

**Background:**

*Blastocystis* is a common anaerobic colonic protist in humans with controversial pathogenicity. *Clostridium difficile* (*C. difficile*) is the commonest cause of infectious diarrhea in healthcare settings. The prevalence and subtype (ST) characteristics of *Blastocystis* in patients with *C. difficile* infection (CDI) are rarely documented. Therefore, the present study was conducted to investigate the prevalence and subtype characteristics of *Blastocystis* in patients with suspicion of CDI in Singapore.

**Methods:**

Fecal samples were collected from 248 patients presenting with suspected CDI from a single tertiary hospital in Singapore. *C. difficile* was diagnosed through positive glutamate dehydrogenase (GDH) with or without toxin A/B using enzyme immunoassay methods. The prevalence and subtype genetic characteristics of *Blastocystis* were determined by polymerase chain reaction (PCR) amplification and analysis of the barcode region of the *SSU* rRNA gene.

**Results:**

The proportion of *C. difficile* in patients with healthcare-associated diarrhea in this study was 44% (109/248). Among the 109 *C. difficile*-positive patients, 59 (54.1%, 59/109) tested positive for toxigenic *C. difficile*, which was considered CDI. Based on the sequence analyses of the barcode region of the *SSU* rRNA gene, 10.1% (25/248) of the patients were found to be *Blastocystis*-positive, and three subtypes were identified: ST7 (64%, 16/25), ST1 (20%, 5/25), and ST3 (16%, 4/25). Remarkably, we found five patients with *Blastocystis* and *C. difficile* coinfection, and further subtype analysis showed two with ST7, two with ST1, and one with ST3.

**Conclusions:**

To the best of our knowledge, this is the first study to investigate the subtype distributions of *Blastocystis* in patients with CDI in Singapore. We found ST7 to be the predominant subtype in diarrheal patients. The pathogenicity of ST7 has been strongly suggested in previous in vitro and mouse model experiments, further confirming its potential pathogenicity to humans.

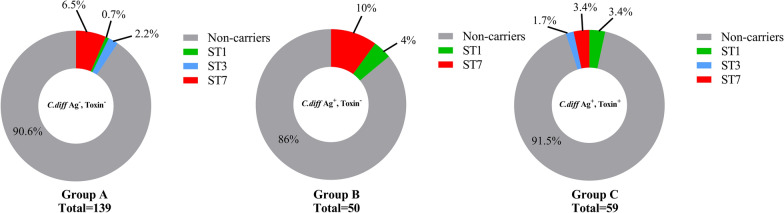

**Supplementary Information:**

The online version contains supplementary material available at 10.1186/s13071-021-04749-8.

## Background

*Blastocystis*, an anaerobic colonic protistan parasite, can colonize the intestines of humans and a wide range of animals [1]. Although *Blastocystis* has been known for more than 100 years, its pathogenicity is still a matter of debate [2]. To date, 22 subtypes have been identified based on analyses of the small subunit (*SSU*) rRNA gene, while ST21 and ST23–26 still need further data to determine [3]. ST1–9 and ST12 were identified in humans, with ST1–4 the most prevalent [4]. A more recent study revealed that ST10 and ST14 are also able to infect humans [5]. The extensive variations in virulence factors and genetic characteristics among *Blastocystis* subtypes have been identified in vitro and in genomic studies [6, 7].

*Blastocystis* has been previously reported to play a central role in modulating the gut microbiota, whereby *Blastocystis* colonization can increase the bacterial diversity and abundance of *Clostridia* [8–10], known producers of short-chain fatty acids, which are usually associated with a healthy gut microbiota [11]. However, other microbiome studies showed that colonization of *Blastocystis* reduced the proportion of beneficial bacteria, such as *Bifidobacterium*, in a mouse model and in patients with irritable bowel syndrome (IBS) [12, 13]. The discrepancy in these results may be due to the enormous genetic variations between different subtypes of *Blastocystis*, which could result in differences in pathogenic potential.

*Clostridium difficile* (*C. difficile*), an important nosocomial pathogen, is the most common causative agent of antibiotic-associated diarrhea [14]. The use of antibiotics in patients with diarrhea can increase the risk of *C. difficile* infection (CDI) by causing dysbiosis [15]. It has been reported that CDI is one of the most common healthcare-associated infections (HAIs) in the United States [16], and CDI can increase in-hospital mortality, prolong hospitalization, and increase medical costs [17]. Pathogenic *C. difficile* strains are armed with multiple toxins, of which the major toxins are TcdA (also known as ToxA) and TcdB (also known as ToxB) [18]. These toxins disrupt the epithelial cell cytoskeleton and cause the disassociation of tight junctions between colonic epithelial cells and loss of epithelial integrity, leading to diarrhea and inflammation in infected patients [19, 20].

To date, there has been only one study reporting the coinfection of *Blastocystis* and *C. difficile* in diarrheal patients from the community and healthcare facilities in Colombia [21]. However, the prevalence of *Blastocystis* and *C. difficile* in diarrheal patients in Singapore is less reported, and the genetic characteristics of different subtypes of *Blastocystis* in CDI patients has yet to be reported. Therefore, this study aims to determine the prevalence of *Blastocystis* and *C. difficile* in patients from a healthcare facility in Singapore, and to further investigate the subtype distributions and genetic characteristics of *Blastocystis* among patients with CDI.

## Methods

### Sample collection

A total of 248 consecutive stool samples from unique individuals were collected from the National University Hospital (NUH) (Additional file 1: Table S1), a tertiary care hospital of 1200 beds, and a major referral center with over 50 medical, surgical, and dental specialties. Sampling was conducted between 2017 and 2019. Samples were only from patients with suspected CDI, defined as having diarrhea arising > 72 h after hospital admission, either receiving or with recent previous antibiotics use, whereby diarrhea was defined as three or more loose stools per day, and defined as type 5 and above on the Bristol stool chart [22]. The study design and protocol were approved by the Domain Specific Review Board of the National Healthcare Group.

### Detection and toxigenic profile of *C. difficile*

The stool samples were first screened for antigens and toxins using the *C. DIFF QUIK CHEK COMPLETE*^®^ kit (D-EIA; TechLab, Blacksburg, VA) according to the manufacturer’s instructions [23]. Briefly, 25 μL of stool specimens was added in a tube containing 750 μL diluent and one drop of conjugate (TechLab). The specimen was mixed either by vortexing or by repeatedly inverting the tube, and then transferred to the device sample well. After incubation for 15 min at room temperature, the wash buffer and the substrate (TechLab) were added to the reaction window. Results were read 10 min later. Glutamate dehydrogenase (GDH) antigen and/or toxins were reported positive if a visible band was seen on the antigen and/or the toxin side of the device display window, respectively. All of the above clinical investigations were conducted by NUH’s Department of Laboratory Medicine, which is accredited by the Singapore Accreditation Council.

### DNA extraction and PCR amplification

Genomic DNA was extracted from stool samples and *Blastocystis* ST7 pure cultures (positive control) using the Qiagen DNA Stool Mini Kit (Qiagen, Hilden, Germany) according to the manufacturer’s instructions. All samples were screened for the presence of *Blastocystis* by polymerase chain reaction (PCR) amplification of the barcode region (a fragment of ~600 bp) of the *SSU* rRNA gene using the primers BhRDr (5′-GAG CTT TTT AAC TGC AAC AAC G-3′) and RD5 (5′-ATC TGG TTG ATC CTG CCA GTA-3′) [24]. Reagents per 25 μl reaction were as follows: 12.5 μl GoTaq^®^ DNA polymerase containing MgCl_2_ (Promega, WI, USA), 1 μl forward primer (0.4 μM), 1 μl reverse primer (0.4 μM), 2 μl genomic DNA, and nuclease-free water to the desired volume. The PCR was started at 94 °C for 4 min followed by 30 cycles of 95 °C for 15 s, 60 °C for 15 s, and 72 °C for 30 s, with an extension at 72 °C for 5 min. Positive DNA (ST7) and negative control (nuclease-free water) was included in all of the PCR tests. The PCR products were subjected to electrophoresis in 1.5% agarose gel (Life Technologies Corporation, CA, USA) stained with SYBR Safe (Life Technologies).

### Detection and subtyping of *Blastocystis*

PCR products with expected fragments (around 600 bp) were subsequently cleaned up using the QIAquick^®^ PCR Purification Kit according to the manufacturer’s instructions (Qiagen, Hilden, Germany) and sent for sequencing (Axil Scientific Pte Ltd, Singapore). Raw sequencing data were checked using Chromas 2.6.6 software (Technelysium, Brisbane, Australia) to guarantee the accuracy of nucleotides. The ambiguous bases at the starts and ends of the sequences were trimmed. The clean sequences then were subjected to BLAST searches (http://www.ncbi.nlm.nih.gov/blast/), and the reference sequences were downloaded from the GenBank database. *Blastocystis* subtypes were identified by BLAST searches (http://blast.ncbi.nlm.nih.gov/Blast.cgi) and the alleles were identified at the *Blastocystis* database (http://pubmlst.org/blastocystis).

### Phylogenetic analysis

A dataset was assembled including the clean sequences obtained in the present study and reference sequences encompassing ST1–17 except for ST11, and an alignment was carried out using the MUSCLE algorithm of MEGA X [25]. The alignment was trimmed using MEGA 6 (http://www.megasoftware.net/), with about a 600-bp barcode region remaining. The barcode region of ST11 is not available, so we excluded ST11 in the phylogenetic analysis [3]. ST21 and ST23-26 were not included because these subtypes require further data for confirmation. The phylogenetic tree was constructed with the neighbor-joining (NJ) method using MEGA 6 software. Evolutionary distances were calculated using the Kimura two-parameter model. The reliability of the trees was assessed by bootstrap analysis with 1000 replicates.

### Statistical analysis

Statistical analyses were performed using SPSS version 22.0 (IBM Corp., Armonk, NY, USA). A chi-square (*χ*^*2*^) test and 95% confidence intervals (CIs) were used to compare the prevalence of *Blastocystis* between different groups. Differences were considered statistically significant at *P*-values < 0.05. Odds ratios (ORs) were also calculated to explore the strengths of association between *Blastocystis* positivity and gender, age, and ethnicity.

## Results

### Prevalence of* C. difficile* in patients with diarrhea

Among the 248 diarrheal stool samples, we found that 139 (56%) samples were negative for *C. difficile* (group A), while 109 (44%) specimens were positive for the *C. difficile* GDH antigen. Furthermore, in terms of the 109 *C. difficile*-positive specimens, 50 (45.9%) of these patients had non-toxigenic *C. difficile* (group B), and 59 (54.1%) tested positive for toxigenic *C. difficile* (group C). It should be noted that CDI was considered only when both the GDH antigen and toxin were positive.

### Prevalence of *Blastocystis* in patients with diarrhea

It is worth noting that since normal PCR was used instead of qPCR, the *Blastocystis* prevalence here refers to the minimum prevalence. In total, *Blastocystis* was detected in 25 of 248 fecal samples (10.1%). Specifically, of the 25 *Blastocystis*-positive patients, 13 were found in group A (9.4%), and seven (14%) and five (8.5%) were identified in group B and group C, respectively (Table 1). The difference in *Blastocystis* prevalence was nonsignificant among different groups (*P* > 0.05).

**Table 1:** The prevalence and subtype distributions of *Blastocystis* among different groups

Groups	No. examined	No. positive	Prevalence (%) (95%)	OR (95%)	*P* value	Subtypes (*n*)
Group A	139	13	9.4% (4.5–14.2)	Reference		ST7 (9), ST3 (3), ST1 (1)
Group B	50	7	14% (4.4–23.6)	1.615 (0.605–4.3110)	0.363	ST7 (5), ST1 (2)
Group C	59	5	8.5% (1.4–15.6)	0.919 (0.312–2.703)	0.844	ST7 (2), ST1 (2), ST3 (1)
Total	248	25	10.1%			ST7 (16), ST1 (5), ST3 (4)

Group A represents *C. difficile* antigen-negative patients; group B represents *C. difficile* antigen-positive and toxigenic-negative patients; group C represents both *C. difficile* antigen- and toxigenic-positive patients

The prevalence of *Blastocystis* in male and female patients was 13.5% and 7.6%, respectively (Table 2), and the difference was not significant (*P* > 0.05). Across age groups, the highest prevalence of *Blastocystis* was observed in ages 18–64 years (13%, 14/108), followed by ≥ 65 years (8.8%, 11/125). *Blastocystis* was not detected in young patients (age < 17 years). Similarly, there was no significant difference in the prevalence of *Blastocystis* among different age groups (*P* > 0.05) (Table 2). The prevalence of *Blastocystis* among various ethnic groups is also presented in Table 2, with the highest prevalence being observed in Indian populations (17.2%, 5/29) and the lowest prevalence in Malay patients (2.6%, 1/39); however, the difference was not significant (*P* > 0.05). The prevalence of *Blastocystis* in antibiotic-treated and untreated patients was 4.5% and 10.6%, respectively, and this difference was also not significant (*P* > 0.05) (Table 2).

**Table 2:** The prevalence of *Blastocystis* in diarrheal patients by gender, age, and ethnicity

Characteristics	No. of examined	No. of positive	Prevalence (%) (95%)	OR (95%)	*P* value
Gender					
Male	104	14	13.5% (6.9–20)	1.881 (0.817–4.33)	0.138
Female	144	11	7.6% (3.3–12)	Reference	
Age (years)					
≤ 17	15	0	0	0	0.999
18–64	108	14	13% (6.6–19.3)	1.554 (0.669–3.56)	0.309
≥ 65	125	11	8.8% (3.8–13.8)	Reference	
Ethnicity					
Chinese	150	16	10.7% (5.7–15.6)	1.075 (0.293–3.945)	0.914
Malay	39	1	2.6% (2.4–7.5)	0.237 (0.023–2.401)	0.223
Indian	29	5	17.2% (3.5–31.0)	1.875 (0.405–8.688)	0.422
Others	30	3	10% (0.7–20.1)	Reference	
Antibiotics					
No indicated	226	24	10.6% (6.6–14.6)	2.495 (0.321–19.386)	0.382
Treated	22	1	4.5% (4.2–13.2)	Reference	
Total	**248**	**25**	**10.1%**		

### Subtype distributions of *Blastocystis* in patients with diarrhea

**Fig. 1:**
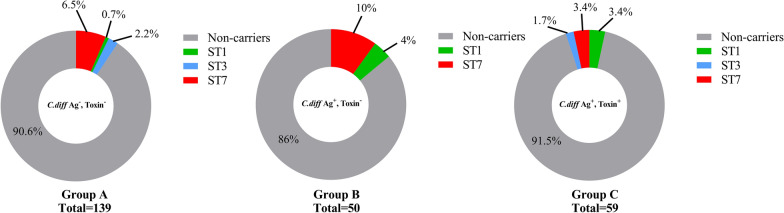
The prevalence and subtype distribution of *Blastocystis* in group A (*C. difficile* negative, and toxin negative), group B (*C. difficile* positive, and toxin negative), and group C (*C. difficile* positive, and toxin positive)

A total of three subtypes (ST1, ST3, and ST7) were identified based on analysis of the barcode region of the *SSU* rRNA gene (Table 1). ST7 was the most common subtype (64%, 16/25), followed by ST1 (20%, 5/25) and ST3 (16%, 4/25). Based on the analysis of *Blastocystis* multilocus sequence typing, for the ST7 isolates four alleles were identified (99, 100, 101, and 137), for ST1 only one allele (4), and for ST3 also one allele (36). Remarkably, we found five patients with *Blastocystis* and *C. difficile* coinfection (Table 1). Subtype analysis revealed two with ST7, two with ST1, and one with ST3, and there was no significant difference in prevalence between different subtypes (*P* > 0.05) (Fig. 1). In contrast, 13 *C. difficile*-negative patients were positive for *Blastocystis* infection, and the prevalence of ST7 (6.5%, 9/139) was significantly higher than ST3 (2.2%, 3/139) and ST1 (0.7%, 1/139) (*P* < 0.05) (Fig. 1).

### Genetic characteristics of *Blastocystis* subtypes

A total of 12 representative sequences were obtained from the 25 *Blastocystis*-positive samples in the present study, including one ST1 sequence, two ST3 sequences, and nine ST7 sequences. The sequence of ST1/allele 4 (MT974098) derived from strains C34, C41, C54, and A37 was identical to that of a *Blastocystis* sample found in sewage water in the Philippines (KY964542). Similarly, the two sequences of ST3/allele 36 (MT974099 and MT974010) obtained from strains C6 and N38 showed 100% identity to that of *Blastocystis* reported from humans in Japan (KT438691 and KT438689, respectively).

ST7 showed high genetic variations within the 600-bp barcode region of *SSU* rRNA of *Blastocystis*. Among the nine ST7 sequences, the sequences of ST7/allele 137 (MT974101) from strains A1, A3, and A4 were identical to the accession number KF447173 identified in a human from France. One ST7/allele 101 sequence (MT974104) derived from strain N31 and one ST7/allele 99 sequence (MT974105) derived from strain N37 showed 100% and 99.35% identity to those in humans in the Czech Republic (MT042799) and Japan (KT438701), respectively. The remaining six ST7 sequences (MT974102, MT974103, and MT974106–MT974109) showed 98.96–99.83% identity with that from humans in France (KF447173).

### Phylogenetic analysis of *Blastocystis*

**Fig. 2:**
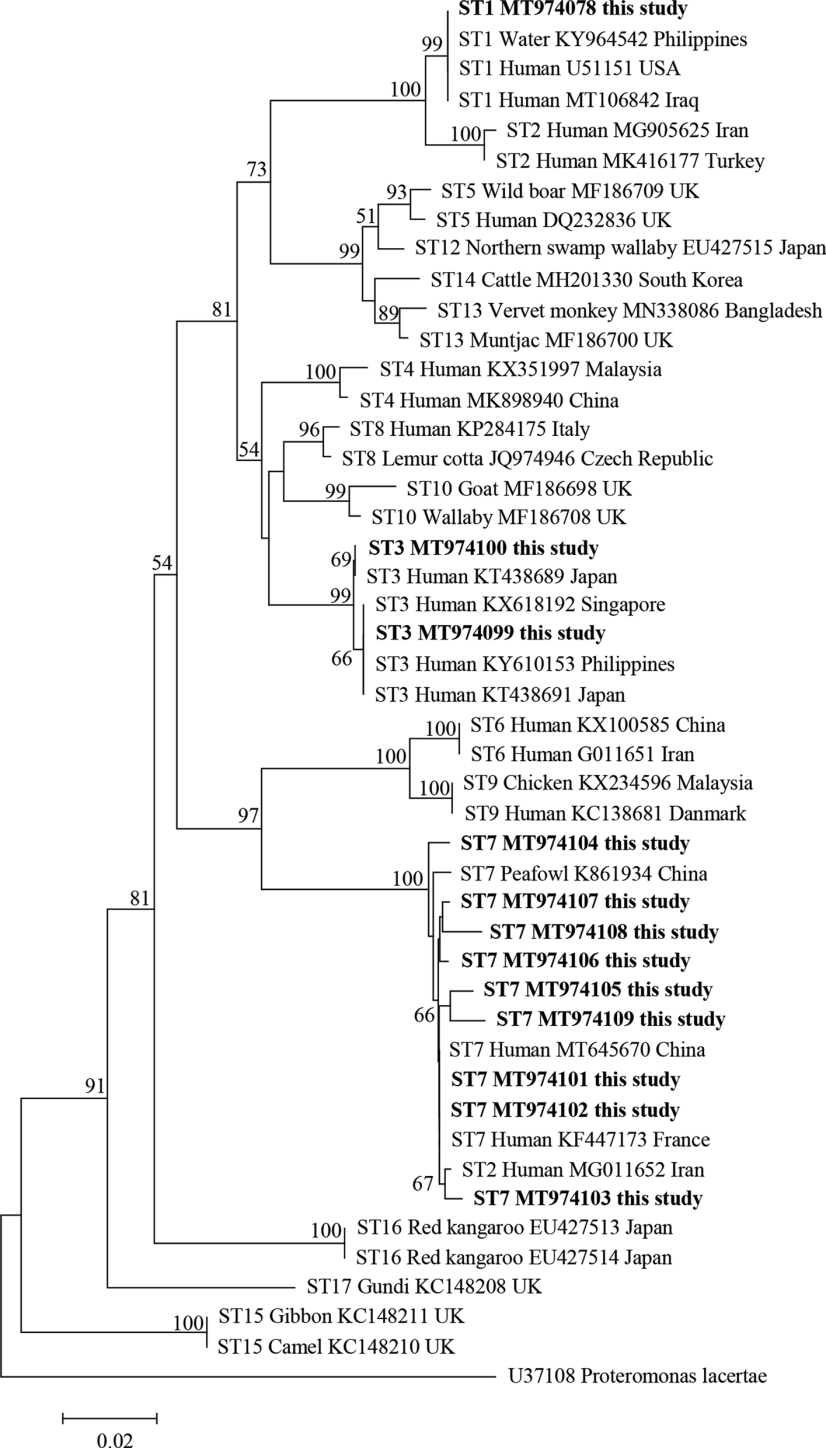
Phylogenetic relationships among nucleotide sequences of *Blastocystis* partial small subunit ribosomal RNA (*SSU* rRNA) gene. The neighbor-joining method was used to construct the trees by the Kimura two-parameter model. The number on the branches are percent bootstrapping values from 1000 replicates, with values of more than 50% shown in the tree. Each sequence is identified by its subtype, host, accession number, and country. *Blastocystis* subtypes identified in the present study are indicated in bold type

The evolutionary relationship of *Blastocystis* subtypes found in the present study was analyzed by the neighbor-joining method. The sequences obtained in this study displayed high identity with *Blastocystis* sequences deposited in GenBank. The phylogenetic tree showed that ST1 was clustered with other ST1 isolated from humans and water samples. ST3 was grouped together with other ST3 identified in humans from different countries. ST7 along with sequences isolated from peafowl and humans clustered together, and formed branches separately (Fig. 2).

## Discussion

The prevalence of *Blastocystis* in humans varies across countries. Generally, the prevalence in developing countries is higher, ranging from 30 to 100%, compared to developed countries (0.5%–30%) [1, 26]. In our study, the prevalence of *Blastocystis* was relatively low, at 10.1%, compared to other developed countries, such as 33.3% in diarrheal patients in the United States [27] and 19% in Australia [28]. However, what is noteworthy is that the prevalence of *Blastocystis* among the diarrheal samples was much higher as compared to an earlier survey on healthy stool samples from NUH in Singapore (3.3%) [29]. It is likely that this difference in prevalence is due to the type of sample collected. In this study, only diarrheal samples were collected, since it is the hallmark of CDI, while the criteria for the collection of fecal samples in the earlier study were not as stringent.

On the other hand, we found that among 22 patients who were on concomitant antibiotics, only one was positive for *Blastocystis*, and of the 25 patients who were positive for *Blastocystis*, only one was on concomitant antibiotics. There are conflicting reports on age as a risk factor for *Blastocystis* infection. However, it is noteworthy that the intestinal microbiota changes associated with age [30] and the use of antibiotics may also influence *Blastocystis* prevalence.

*C. difficile* is commonly known as a hospital- and antibiotic-associated pathogen and can causes life-threatening diarrhea and colitis [31]. The overall proportion of toxigenic *C. difficile* (23.8%, 59/248) from this study was lower than that reported in diarrheal patients from Colombia (57.3%) [21], but higher than findings in China (14%) [32] and an early survey in unformed stool samples from Singapore General Hospital (SGH) in Singapore (9.5%) [33]. Similarly, the proportion of toxigenic *C. difficile* was 9.6% (158/1642) from NUH in Singapore [34], and the proportion was 12.5% (276/2212) by both *C. difficile* toxin assay (CDTA) and International Classification of Diseases, Ninth Revision (ICD-9) codes at Tan Tock Seng Hospital in Singapore [35]. Indeed, the prevalence of CDI has increased dramatically worldwide in recent years, especially in Europe and North America [14]. The possible reasons are the widespread use of broad-spectrum antibiotics and the increased prevalence of IBD among others. In addition, the type of test may also affect the prevalence of *C. difficile*. We used enzyme immunoassay to detect the antigen of *C. difficile*, which is an initial test due to its high diagnostic sensitivity, while Vega et al. applied PCR tests with high sensitivity and specificity. This may be one of the reasons for the difference in prevalence found among studies.

Vega et al. reported a significant association between the presence of *Blastocystis* and CDI in patients with diarrhea [21]. However, we did not find a significant correlation, with only five patients found to be coinfected. Indeed, several clinical studies have indicated that CDI is associated with dysbiosis, and can increase the oxygen content in the intestine [36]. It has been determined that high concentrations of oxygen can affect *Blastocystis* colonization in the context of dysbiosis [37]. On the other hand, growth of certain facultative anaerobes could also result in high oxygen concentration [38], which may have a detrimental effect on *Blastocystis* colonization due its anaerobic nature.

Interestingly, the present study revealed that a rare subtype (ST7) was the most prevalent in diarrhea stool samples. A well-studied, pathogenic isolate of ST7 was originally isolated from a patient with gastrointestinal symptoms in Singapore [39], but it has subsequently been identified in humans in many countries, with prevalence ranging from 0.8% in Nigeria [40] to 17.9% in Thailand [41]. ST7 has been suggested to be a pathogenic subtype based on in vitro and in vivo mouse studies. It was found to decrease the abundance of beneficial *Bifidobacterium* and *Lactobacillus* in a DSS-induced colitis mouse model, leading to a dysbiotic state [12]. Additionally, the cystine proteases produced by ST7 can compromise tight junction proteins zonula occludens-1 (ZO-1) and F-actin in vitro, thereby increasing the permeability of intestinal epithelial cells [42, 43]. The present study reported high prevalence of ST7 in diarrheal patients, but we were not provided protected health information, and were thus unable to match leukocyte counts and serum creatinine to determine CDI severity, which is a shortcoming of this study.

In this study, considerable intra-ST genetic polymorphisms were found in ST7, and four alleles (allele 99, allele 100, allele 101, and allele 137) were observed in ST7. A more recent study reported ST7 alleles 41, 106, 110, and 112 in gut-healthy humans in the Czech Republic [44]. ST7 was also found in Indian peafowl in China [45], and ST7 (allele 99) was reported in companion animals in a recent study [46], suggesting that ST7 can be transmitted between domestic animals and between animals and humans.

ST1 and ST3 are two common subtypes in humans [47], and are usually associated with healthy gut microbiota. Indeed, these two subtypes are usually identified in asymptomatic patients and can colonize for long periods without any clinical symptoms [48]. Next-generation sequencing (NGS) of the 16S rRNA gene carried out on 2524 subjects in Italy revealed that *Blastocystis* ST3 carriers were associated with high bacterial diversity and potentially beneficial species such as *Prevotella* and *Ruminococcus* [49]. Similarly, ST1- and ST3-colonized individuals are mainly associated with *Prevotella* and *Ruminococcus* enterotypes and linked to higher bacterial richness [50]. Notably, Terveer et al. used *Blastocystis*-positive (ST1 and ST3) donor samples to treat recurrent *Clostridioides difficile* infections (rCDI) through fecal microbiota transplantation (FMT), and demonstrated that the presence of *Blastocystis* ST1 and ST3 from donors did not cause any adverse gastrointestinal symptoms or have any significant effect on treatment outcome [51]. Although ST1 and ST3 were also found in this study, their prevalence was very low (3.6%). More research is needed to better understand the role of *Blastocystis* ST1 and ST3 in diarrheal patients.

## Conclusions

The present study determined that the prevalence of *Blastocystis* and the proportion of *C. difficile* from clinical samples at a local hospital (NUH) were 10.1% and 43.4%, respectively. Molecular analysis identified three subtypes (ST1, ST3, and ST7), with the geographically rare ST7 as the predominant subtype. Our current study supports the idea that ST7 is a potentially pathogenic subtype, and future research should focus on its relationship with gastrointestinal symptoms and its role in the host intestinal microbiota.

## Supplementary Information

**Additional file 1: Table S1.** Detailed information about each patient.

## Data Availability

The nucleotide sequences generated in present study have been deposited in GenBank (https://www.ncbi.nlm.nih.gov/) under accession numbers MT974098–MT974109.

## Abbreviations

*C. difficile*: *Clostridium difficile*
PCR: Polymerase chain reaction
ST: Subtype
*SSU* rRNA: Small subunit ribosomal RNA
IBS: Irritable bowel syndrome
CDI: *C. difficile* Infection
HAIs: Healthcare-associated infections
NUH: National University Hospital
BLAST: Basic Local Alignment Search Tool
ORs: Odds ratios
SGH: Singapore General Hospital
CDTA: *C. difficile* toxin assay
ICD-9: International Classification of Diseases, Ninth Revision
ZO-1: Zonula occludens-1
rCDI: Recurrent *Clostridioides difficile* infections
FMT: Fecal microbiota transplantation
